# Can tweets be used to detect problems early with scientific papers? A case study of three retracted COVID-19/SARS-CoV-2 papers

**DOI:** 10.1007/s11192-021-03962-7

**Published:** 2021-04-26

**Authors:** Robin Haunschild, Lutz Bornmann

**Affiliations:** 1 Max Planck Institute for Solid State Research, Heisenbergstr. 1, 70569 Stuttgart, Germany; 2 Science Policy and Strategy Department, Max Planck Society, Administrative Headquarters, Hofgartenstr. 8, 80539 Munich, Germany

**Keywords:** Twitter, Scientometrics, Altmetrics, COVID-19, SARS-CoV-2, Retracted papers

## Abstract

Methodological mistakes, data errors, and scientific misconduct are considered prevalent problems in science that are often difficult to detect. In this study, we explore the potential of using data from Twitter for discovering problems with publications. In this case study, we analyzed tweet texts of three retracted publications about COVID-19 (Coronavirus disease 2019)/SARS-CoV-2 (severe acute respiratory syndrome coronavirus 2) and their retraction notices. We did not find early warning signs in tweet texts regarding one publication, but we did find tweets that casted doubt on the validity of the two other publications shortly after their publication date. An extension of our current work might lead to an early warning system that makes the scientific community aware of problems with certain publications. Other sources, such as blogs or post-publication peer-review sites, could be included in such an early warning system. The methodology proposed in this case study should be validated using larger publication sets that also include a control group, i.e., publications that were not retracted.

## Introduction

Certain manuscripts are retracted after publication, although they have been reviewed by colleagues in editorial peer reviews (Sotudeh et al., 2020). There are different reasons for retractions of publications. The main reasons are data errors, methodological mistakes, and, or scientific misconduct. These errors can further be “distinguished between ‘reputable’ errors (those arising despite the investigators’ best efforts to avoid them) and ‘disreputable’ errors (those arising from disregard of acceptable scientific practice).” (Zuckerman, 2020, p. 954) Fang et al., (2012) reported that “2,047 biomedical and life-science research articles indexed by PubMed as retracted on May 3, 2012 revealed that only 21.3% of retractions were attributable to error. In contrast, 67.4% of retractions were attributable to misconduct, including fraud or suspected fraud (43.4%), duplicate publication (14.2%), and plagiarism (9.8%).” An overview of definitions, manifestations, and extent of research misconduct can be found in Bornmann (2013).

In cases of minor problems with a published paper, a correction is usually published. However, if correcting the problems alters conclusions of the study, retraction can be the only possibility for correcting the scientific record. Retraction decisions are usually not done with levity because retractions *damage the scientific reputation of authors and journals*. Retractions may cast a poor light on the research capacity of authors and the functionality of the editorial peer review. Therefore, one should expect that retractions do not occur very often. However, occurrences of scientific misconduct seem to become known to the scientific public often enough so that the topic of scientific misconduct has become “a thriving area of research” (Zuckerman, 2020, p. 945). The Web of Science (WoS; Birkle et al., 2020) has a document type named “retraction” that has indexed documents with publication years since 1974.

Rumors about data errors, methodological mistakes, and scientific misconduct can spread very quickly on social media such as Twitter (a popular micro-blogging platform, see https://www.twitter.com) (da Silva Jaime & Dobránszki, 2019). Sugawara et al., (2017) found that discussions on scientific misconduct regarding a study on stimulus-triggered acquisition of pluripotency (STAP) cells in Japan in 2014 occurred on Twitter before they surfaced in newspapers. Retracted publications obtain fewer citations either before or after retraction than non-retracted publications (Sotudeh et al., 2020). This is rather different for Twitter counts. Retracted publications may become more frequently mentioned on Twitter than ever due to their retraction (Bornmann & Haunschild, 2018). Thus, Twitter counts may not reflect the quality of publications.

Do Twitter users mention problems with papers (early) that are later retracted? Are Twitter users who mention retracted publications informed about the paper status? Do they explicitly mention the issues why the publication has been retracted? These are the questions we try to answer in this case study by analyzing the tweets that mention three different retracted publications about COVID-19 (Coronavirus disease 2019)/SARS-CoV-2 (severe acute respiratory syndrome coronavirus 2) and their retraction notices. Since Twitter is a very fast medium, tweets might be used as early indicators for problems with certain publications.

The World Health Organization (WHO) declared the 2019–2020 coronavirus outbreak a Public Health Emergency of International Concern (World Health Organization, 2020) on 30 January 2020 and a pandemic on 11 March 2020 (Ghebreyesus, 2020). We selected the three publications from a post from Retraction Watch about retracted COVID-19/SARS-CoV-2 papers (https://retractionwatch.com/retracted-coronavirus-covid-19-papers/). As of 24 September 2020, Retraction Watch lists 33 COVID-19/SARS-CoV-2 papers as retracted. We selected the papers for this case study with the requirements that there is a Digital Object Identifier (DOI) and known publication date for the publication and its retraction notice and the dates of publication and retraction are at least 2 weeks apart:

1. Bae et al., (2020a) studied the effectiveness of surgical and cotton masks in blocking SARS–CoV-2. The study was published on 6 April 2020 and retracted on 2 June 2020 because they “had not fully recognized the concept of limit of detection (LOD) of the in-house reverse transcriptase polymerase chain reaction used in the study” (Bae et al., 2020b). In this case, the retraction was made because of a methodological error that was not detected in the peer review process.
2. Wang et al., (2020a) reported that “SARS-CoV-2 infects T lymphocytes through its spike protein-mediated membrane fusion”. The peer review process was very fast: the paper has been submitted on 21 March 2020 and accepted 3 days later on 24 March 2020. This paper has been published on 7 April 2020, and retracted on 10 July 2020 (Wang et al., 2020b) because “[a]fter the publication of this article, it came to the authors attention that in order to support the conclusions of the study, the authors should have used primary T cells instead of T-cell lines. In addition, there are concerns that the flow cytometry methodology applied here was flawed. These points resulted in the conclusions being considered invalid.” In this case, the retraction was made because of methodological errors that were not discovered during the peer review process.
3. Probably the most attention among the three publications was drawn to the study by Mehra et al., (2020a). They reported that they could not confirm a benefit in COVID-19 treatment with hydroxychloroquine. They even reported that hydroxychloroquine increases the risk of complications during medical treatment against COVID-19. The study was published on 22 May 2020, and retracted on 05 June 2020 (Mehra et al., 2020a, b) because “several concerns were raised with respect to the veracity of the data and analyses conducted by Surgisphere Corporation and its founder” and co-author of the study. Surgisphere declined to transfer the full dataset to an independent third-party peer reviewer because that would violate client agreements and confidentiality requirements. Potential benefit or risk of hydroxychloroquine for treatment of COVID-19 is still not clear. In this case, the retraction was made because of doubts regarding the validity of the employed data that was not discovered in the peer review process.

These three publications have received considerable attention in online media as can be seen from their Altmetric Attention Scores. Costas et al., (2015) studied the relation between various bibliometric and altmetric indicators and found that the Altmetric Attention Score mainly correlates with Twitter counts. On 13 August 2020, we consulted the altmetrics detail website by Altmetric.com for the three publications. Despite very different Altmetric Attention Scores (25,175, 7254, and 3357) all three publications range in “the top 5% of all research outputs scored by Altmetric”. The Altmetric Attention Score “is a weighted count of all of the mentions Altmetric has tracked for an individual research output, and is designed as an indicator of the amount and reach of the attention an item has received” (https://www.altmetric.com/blog/the-altmetric-score-is-now-the-altmetric-attention-score/, last accessed on 3rd of February 2021).

## Short literature review about studies regarding retracted publications

In this short literature review, we focus on very recent publications dealing with retracted papers and those that additionally analyze the relation between retracted papers and altmetrics, in particular tweets. In the latter case, the literature is very sparse.

Vuong et al., (2020) analyzed 18,603 retractions of publications using data from Retraction Watch. The earliest paper was published in 1753 and retracted in 1756. The longest time period between publication and retraction was 80 years. Retractions increased dramatically since 2000 reaching more than 1000 retractions in 2009 and anomalously high 4867 retractions in 2010. The countries with the most retracted publications were China (*n* = 8612) and USA (*n* = 3179) followed by India (*n* = 934) and Japan (*n* = 874). Similar retraction frequencies were reported by Zhang et al., (2020). These authors also found a negative correlation between proportions of international collaborations and retraction rates.

Roe and Lewison (2019) analyzed publications with the term “retracted” in their titles and published between 1998 and 2017. They found 5566 retracted papers in this time period (0.02% of the WoS database content). A spike of retractions was found in 2011 that “was caused by the withdrawal of all 762 of the papers in the printed volume of the 2011 International Conference on Energy and Environmental Science (ICEES, 2011), Energy Procedia, Volume 11. The conference was held in Singapore, and was “removed by the publisher due to insufficient assurances by the programme organisers that the professional ethical codes of publishing and standards were applied consistently (https://www.sciencedirect.com/science/article/pii/S1876610214004536).” (Roe & Lewison, 2019, p. 60) Rathmann and Rauhut (2019) analyzed 3549 retracted publications with the same number of non-retracted publications (as a control group). They found that larger teams show a lower probability of having co-authored a retracted publication than smaller teams. Thus, it seems that collaboration might prevent methodological mistakes, data errors, and scientific misconduct.

Some studies analyzed retracted publications in various fields. Rapani et al., (2020) analyzed 180 retracted publications from the dental literature that were published between 2001 and 2018. They found an increase of 47% of retractions within the time period 2014–2018. The main reason for retraction was scientific misconduct followed by honest scientific mistakes (i.e., reputable errors). About half of the retracted publications contained at least one co-author from Asia (Rapani et al., 2020). Bar-Ilan and Halevi (2019) studied the pre- and post-retraction citations of 3435 papers from the Retraction Watch database. They found that retracted papers still receive citations but on average much less after than before retraction. Chambers et al., (2019) studied 176 retracted papers in the fields of obstetrics and gynecology. The most frequent reasons for retractions in these cases were scientific misconduct and data errors. Rosenkrantz (2016) analyzed 48 retracted publications from radiology journals. One third of the publications was retracted because of methodological errors. Erfanmanesh and Teixeira da Silva (2019) analyzed the retraction rate of 16 open access mega journals in the time period 2012–2018. Nine of them did not show a single retraction. The three open access mega journals with the highest retraction rates were found to be *Medicine*, *Cell Reports*, and *PLOS ONE*. They casted doubt on the efficacy of the soundness of the peer review model at open access mega journals.

We found only a few studies that used altmetrics data to investigate retracted publications. Haustein et al., (2015) reported that retracted publications were more frequently mentioned in tweets and blogs than average publications. Copiello (2020) compared 209 retracted publications to 418 contemporary, non-retracted publications from *PLOS ONE.* They reported that frequencies of mentions of publications on post-publication peer-review sites, such as PubPeer, significantly deviated from the average for the retracted publications. Bornmann and Haunschild (2018) reported that a rarely tweeted publication before retraction received 83.6% of its mentions on Twitter after retraction and therefore casted doubt on Twitter as a source for research evaluation. Yuan et al., (2019) analyzed different kinds of impact (scientific, technological, funding, and altmetrics) of 39 retracted publications from the journal *Cell*. They found medium correlations (values between 0.3 and 0.5 of Pearson's correlation coefficient) between scientific, technological, and altmetrics impact, while a negligible correlation (Pearson's correlation coefficient below 0.2) between funding impact and the other kinds of impact was found. Shema et al., (2019) analyzed the relationship between reasons of retractions and citation-based indicators and the Altmetric Attention Score. They found that “retraction because of misconduct triggers a further increase of altmetric attention for publications which already accumulated altmetric attention before.” (p. 107).

Whereas the few studies using altmetrics data to investigate retracted publications are based on quantitative-statistical approaches for analyzing retracted publications, we apply in this study a qualitatively oriented approach to find indications of problems with publications in tweets.

## Methods

We used the Altmetric.com application programming interface (API) for extracting tweet identifiers for any tweets that mentioned either any of the three retracted publications or their retraction notices on Twitter. The tweet IDs of the tweets that mentioned any of the publications or retractions on Twitter were downloaded from the Twitter API. We connected to both APIs using R (R Core Team, 2019) with the R packages httr (Wickham, 2017a) and RCurl (Lang & the CRAN team, 2018). The tweets were downloaded from the 6th to the 9th of August, 2020 via the Twitter API using R (R Core Team, 2019) and stored in local SQLite database files using the R package RSQLite (Müller et al., 2017). Functions from the R package DBI were used for sending database queries (R Special Interest Group on Databases (R-SIG-DB), Wickham, & Müller, 2018).

The R package ggplot2 was used for plotting the time evolution of tweets (Wickham, 2016). The R package tidyverse (Wickham, 2017b) was used for the analyses of the Twitter user profiles. The R package UpSetR (Gehlenborg, 2019) was used for plotting classifications of Twitter user profiles. The R packages tm (Feinerer et al., 2008), NLP (Hornik, 2014), SnowballC (Bouchet-Valat, 2014), wordcloud (Fellows, 2014), and RColorBrewer (Neuwirth, 2014) were used for producing word clouds. Before searching in tweet texts and displaying them as word clouds, the tweet texts were converted into lower case characters and only alpha-numeric characters were kept. English stop words, punctuation characters, and needless whitespaces were removed in order to obtain more robust word clouds.

**Fig. 1:**
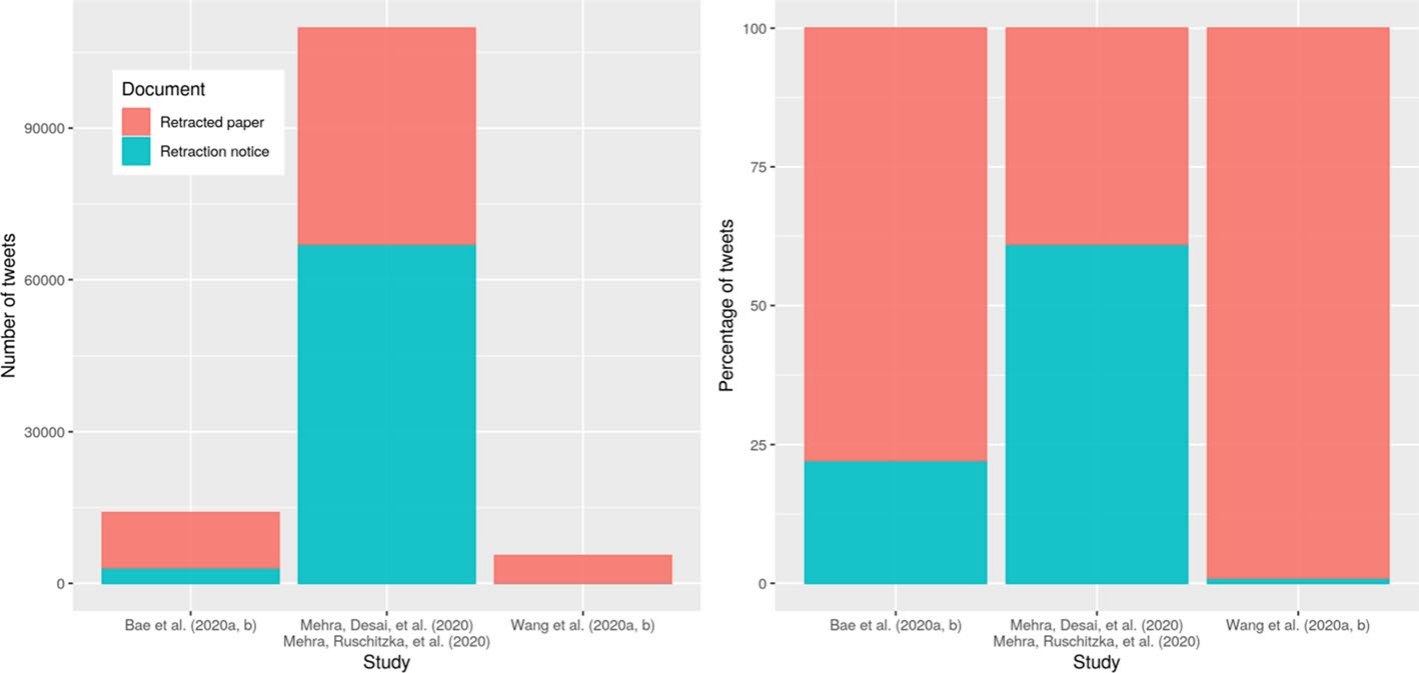
Number (left panel) and percentage (right panel) of tweets mentioning any of the three publications or their retraction notices

We downloaded 42,746 tweets for Mehra et al., (2020a) and 67,038 tweets for its retraction notice; 10,875 tweets for Bae et al., (2020a) and 3095 tweets for its retraction notice; 5428 tweets for Wang et al., (2020a) and 53 tweets for its retraction notice. Figure 1 shows the number and percentage of tweets mentioning each of the publications and their retraction notices. The results show that the retractions have received very different numbers of tweets compared to the corresponding papers.

In order to receive information about the people mentioning the three publications or their retraction notices in tweets, we used the classification scheme proposed by Toupin et al., (2019) (see Table 1) and a modified version of the R code provided by Toupin (2020). The general idea behind most classifications is to capture the interest of a particular account to share scholarly papers using self-descriptions in the profiles (see Haustein, 2019; Toupin & Haustein, 2018; Vainio & Holmberg, 2017). Note that one Twitter user account may be assigned to multiple classifications.

**Table 1:** Twitter user classification scheme proposed by Toupin et al. (2019)

User	Profile
Faculty and students	Higher education or the realm of research
Communicators and journalists	Transmission of information at higher scale (e.g., media, arts, literature)
Professionals	Engaging with research publications relevant to their job (e.g., conservation manager)
Political	Engaging with research publications with political interest (e.g., through activism or as part of governmental jobs)
Personal	Self-describe themselves using personal interests (e.g., in cats or dogs)
Institutions and organizations	Represent a group of people
Bots	Use keywords related to automated activity
Journals and publishers	Represent journals or scientific publishers

**Fig. 2:**
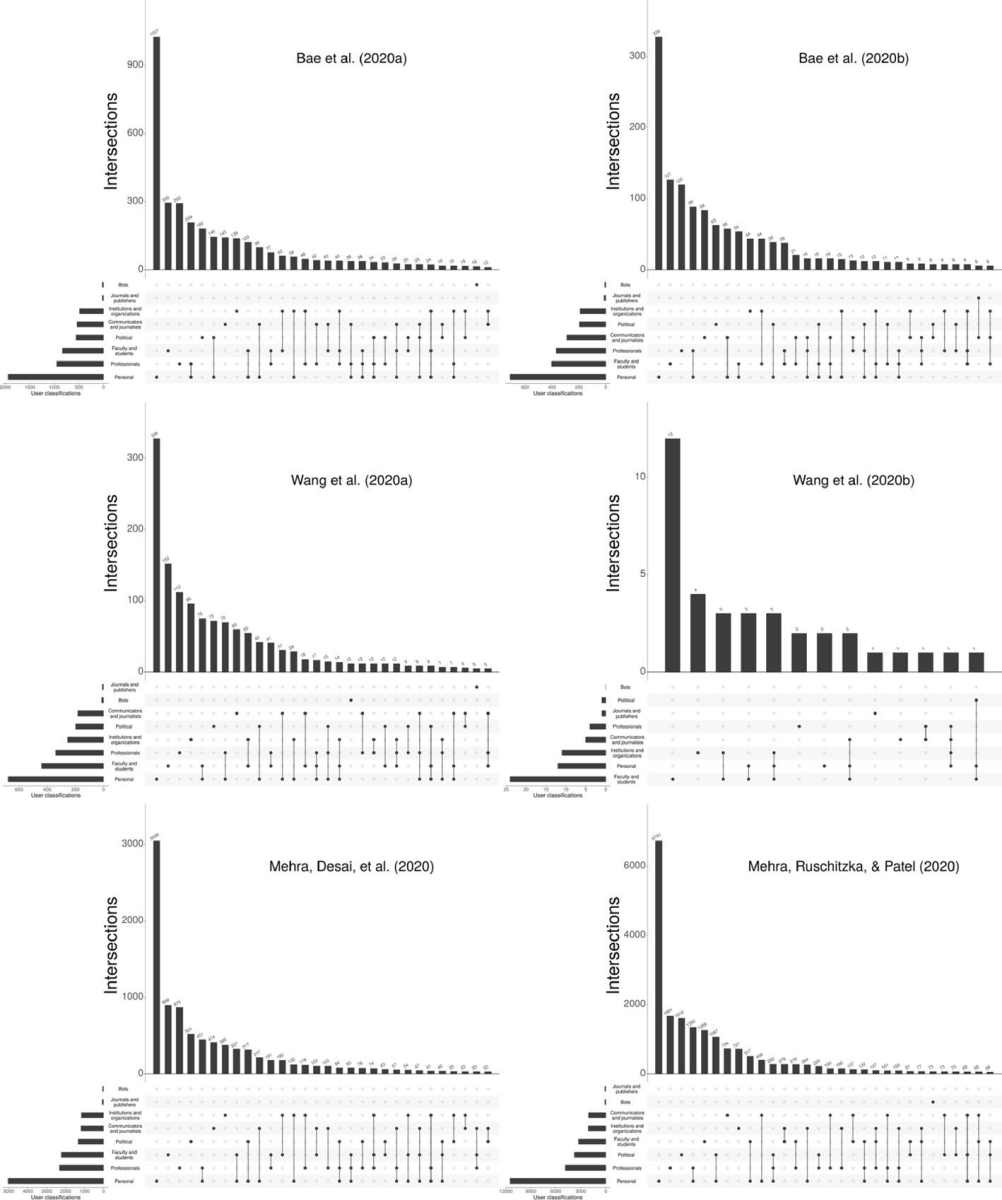
Twitter classification of users mentioning any of the three publications or their retraction notices in tweets

Figure 2 shows the twitter user classifications for all six documents (three retracted papers and their retraction notices) of this case study. The bars on the left of each graph reveal the frequencies of the different Twitter user classifications. The bars to the top of each graph show the frequencies of the most commonly occurring Twitter user classification combinations. The combination type is indicated by the black dots in the lower part of each graph. Combinations have connection lines between the dots. For all documents except Wang et al., (2020b), personal accounts are the largest single group. In the case of Wang et al., (2020b), the user group ‘faculty and students’ is the largest single group. The user groups ‘bots’ and ‘journals and publishers’ are the least frequently occurring groups for all six documents. We expect the most frequent hints to problems with papers especially from the user group ‘faculty and students’ since this group includes people with the necessary expertise. A critical discussion of papers can be scarcely expected from the user groups ‘bots’ and ‘journals and publishers’.

## Results

### Twitter activity of Bae et al., (2020a) and (2020b)

**Fig. 3:**
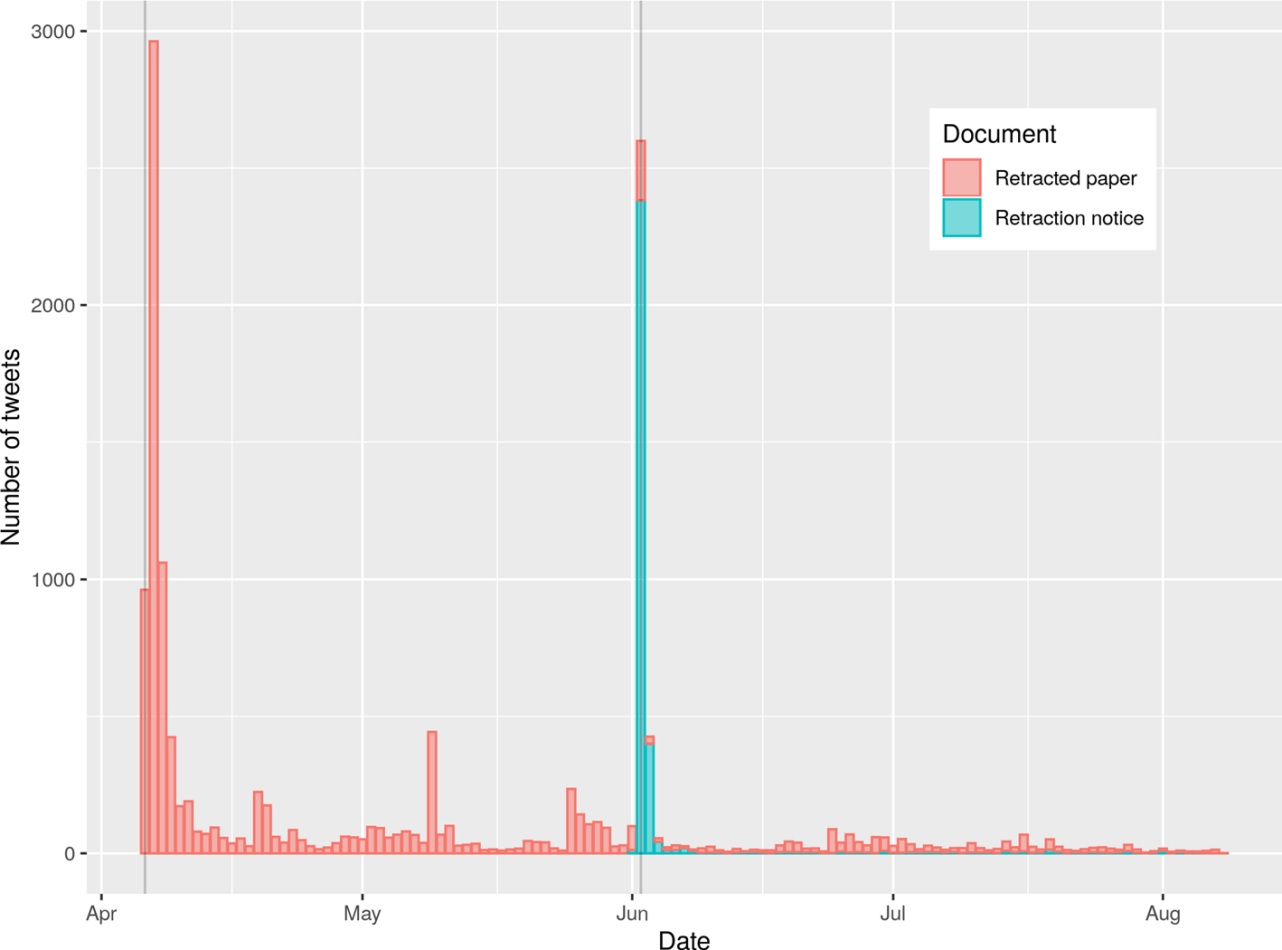
Tweets per day that mentioned either the paper Bae et al., (2020a) or its retraction Bae et al., (2020b). The publication dates of the paper and the retraction notice are marked as gray vertical lines

Figure 3 shows the tweets per day that mentioned either the paper Bae et al., (2020a) or its retraction Bae et al.,(2020b). The publication dates of the paper and retraction notice are marked as gray vertical lines. Most tweets mentioning the publication occurred before retraction. However, the second-most frequent tweets per day were recorded on the day of retraction. Therefore, the tweet texts are analyzed using two different time periods: (i) before the publication date of the retraction and (ii) since the publication date of the retraction.

**Fig. 4:**
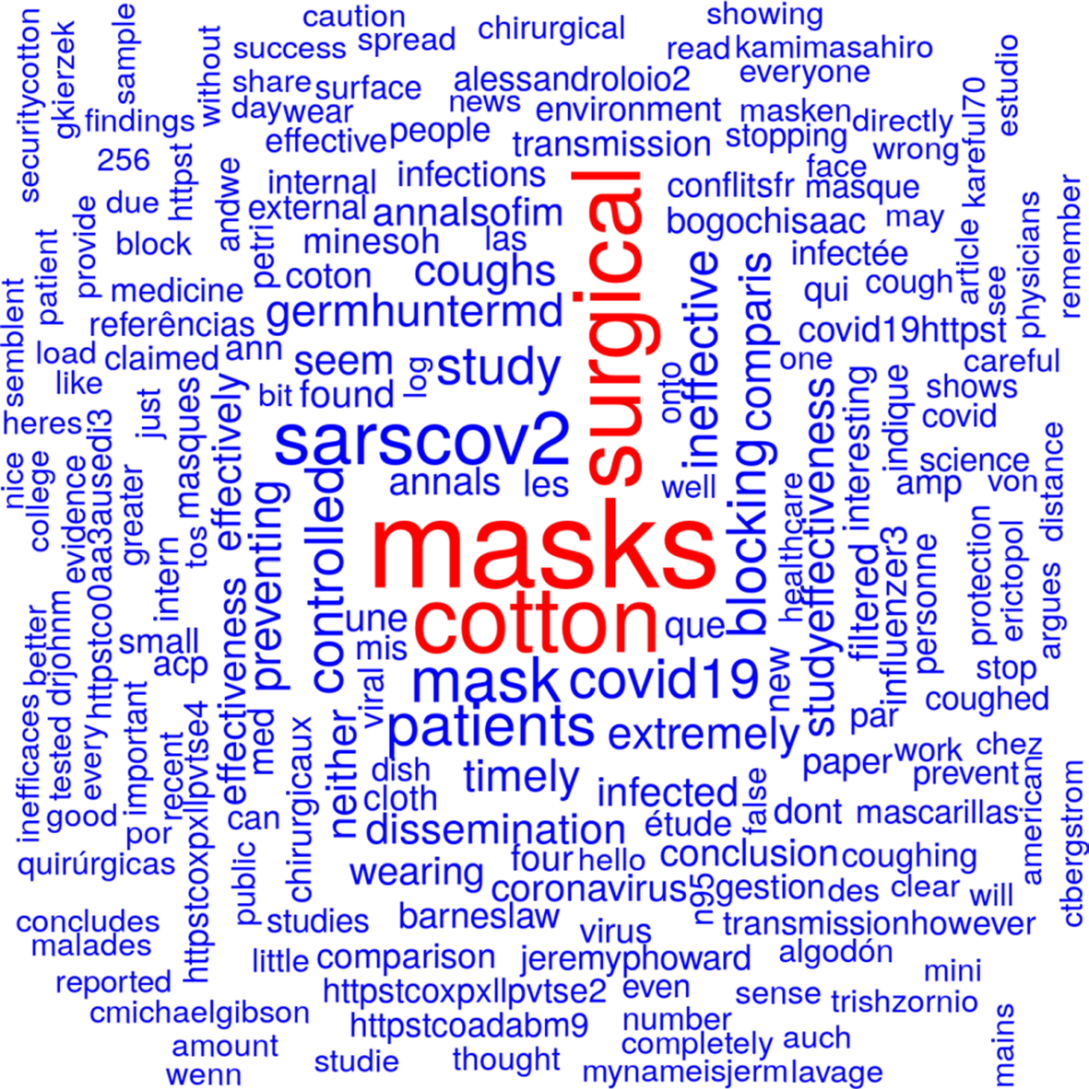
Word cloud from tweet texts based on the tweets mentioning Bae et al., (2020a) before publication date of the retraction

**Fig. 5:**
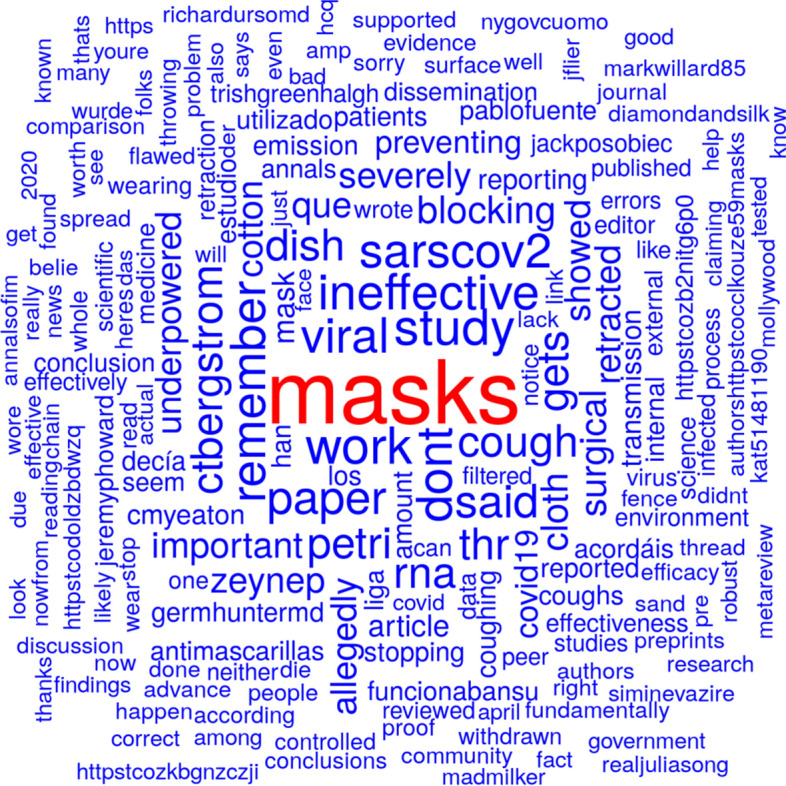
Word cloud from tweet texts based on the tweets mentioning Bae et al., (2020a) or Bae et al., (2020b) since publication date of the retraction

Figure 4 shows a word cloud from tweet texts based on the tweets mentioning Bae et al., (2020a) before publication date of the retraction. Figure 5 shows a word cloud from tweet texts based on the tweets mentioning Bae et al., (2020a) or Bae et al., (2020b) since publication date of the retraction. In both word clouds, the term ‘mask’ is the most prominent one. The terms 'surgical' and 'cotton' are the second- and third-most prominent ones in Fig. 4. The two terms are much less prominent in Fig. 5 although all three terms (‘masks', 'cotton', and 'surgical') appear in the title of both publications.

The publication was retracted because a limit of detection (LOD) has not been recognized. The terms ‘LOD' or 'limit of detection' cannot be spotted on either Figs. 4 or 5. In fact, the term ‘LOD' does not appear on the tweets before retraction and only between 19 times since retraction. The term ‘limit of detection' occurred once before retraction (on the evening before where it probably has been announced already) and seven times after retraction. Three tweets mentioned both, ‘LOD' and 'limit of detection'. In the case of Bae et al., (2020a), we can conclude that the reason for retraction has not been mentioned by Twitter users before a retraction decision has been made and only very rarely (0.5%) after publication of the retraction.

### Twitter activity of Wang et al., (2020a) and (2020b)

**Fig. 6:**
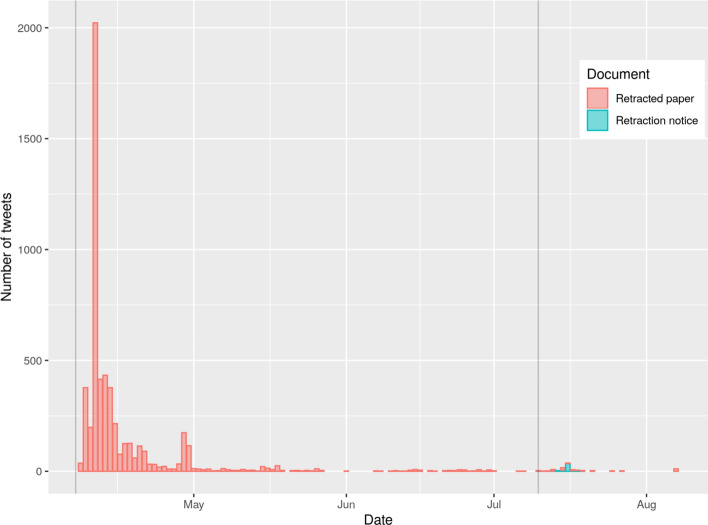
Tweets per day that mentioned either the paper Wang et al., (2020a) or its retraction Wang et al., (2020b). The publication dates of the paper and the retraction notice are marked as gray vertical lines

Figure 6 shows the tweets per day that mentioned either the paper Wang et al., (2020a) or its retraction Wang et al., (2020b). The publication dates of the paper and the retraction notice are marked as gray vertical lines. Most tweets mentioning the publication occurred before retraction. Only very few tweets mentioned the retraction notice (*n* = 53) or the paper after retraction (*n* = 50). The tweet texts are analyzed using two different time periods: (i) before the publication date of the retraction and (ii) since the publication date of the retraction.

**Fig. 7:**
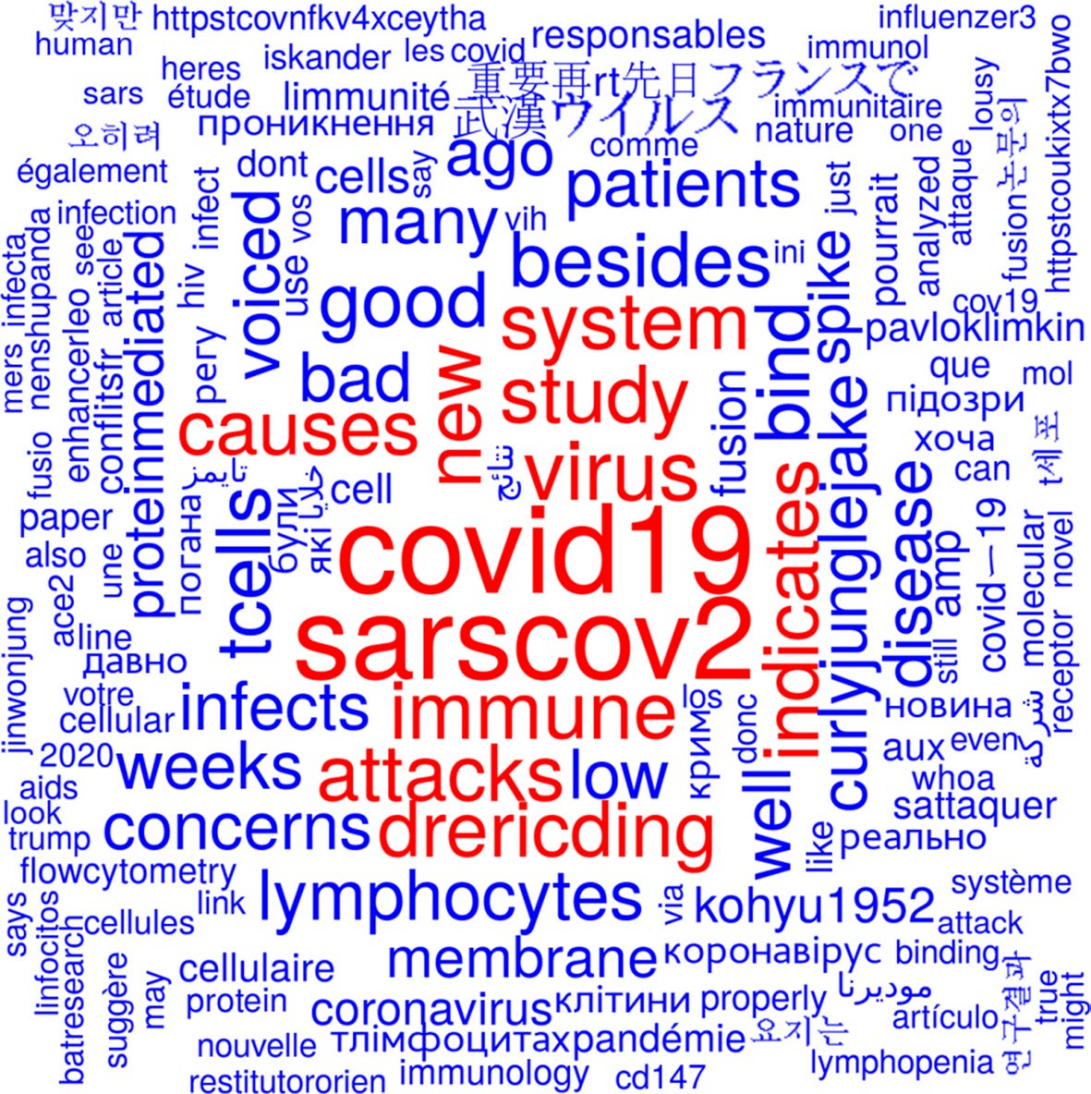
Word cloud from tweet texts based on the tweets mentioning Wang et al., (2020a) before publication date of the retraction

**Fig. 8:**
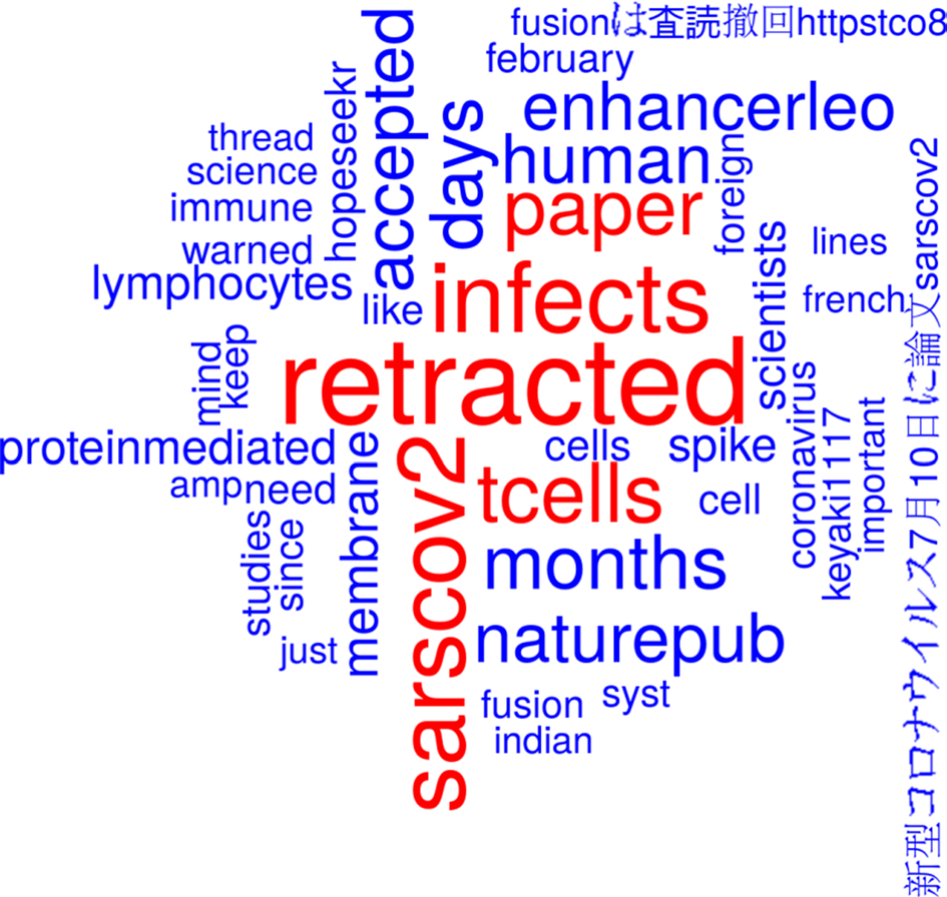
Word cloud from tweet texts based on the tweets mentioning Wang et al., (2020a) or Wang et al., (2020b) since publication date of the retraction

Figure 7 shows a word cloud from tweet texts based on the tweets mentioning Wang et al., (2020a) before the publication date of the retraction. Figure 8 shows a word cloud from tweet texts based on the tweets mentioning Wang et al., (2020a) or Wang et al., (2020b) since the publication date of the retraction. The most prominent terms in Fig. 7 seem to be related to the virus and the disease it is causing whereas the term ‘retracted' is the most prominent one in Fig. 8.

One can easily see the terms ‘tcells’ in Figs. 7 and 8, although not being among the most prominent terms. The study Wang et al., (2020a) has been retracted because the authors should have used primary T cells instead of T-cell lines and because the flow cytometry methodology applied in the study was flawed. There were 5378 tweets registered before retraction and 103 since then. Only 14 tweets before retraction and four since then included the term ‘primary’ in the tweet text. The first tweet that mentioned problems with the used T cells was posted on 9 April 2020 (i.e., 2 days after publication and about 3 months before retraction of the study) and it reads: “I would take this with a grain of salt. This was on cell lines, not primary T-cells. No evidence that this infection results in T-cell dysfunction. Furthermore, severe disease is characterized by hyper-immune response. Much is left to be desired here. Not sure what to conclude. https://t.co/15qvCa0fvw” (Sidholm, 2020).

There were 23 tweets that contained the term ‘flow cytometry’ and 101 tweets that contained the term ‘flowcytometry’ (mainly as a hashtag), both before publication of the retraction notice. Most of these 124 tweets (*n* = 117) were retweets as identified by their first two letters ‘RT’ followed by a whitespace. The first tweet that casted doubt on the employed flow cytometry methodology was posted on 9 April, 2020 and it reads “Lots of discussion on Twitter about this paper, however some questions outstanding: Does the flow cytometry actually show cell infection? Where is the positive control comparison using classically infected cells?” (Vincent, 2020) Only a single tweet (retweeted 26 times) was found that mentioned ‘flowcytometry’ (as a hashtag) and none that mentioned ‘flow cytometry’ after publication date of the retraction notice.

### Twitter activity of Mehra et al., (2020a), and (2020b)

**Fig. 9:**
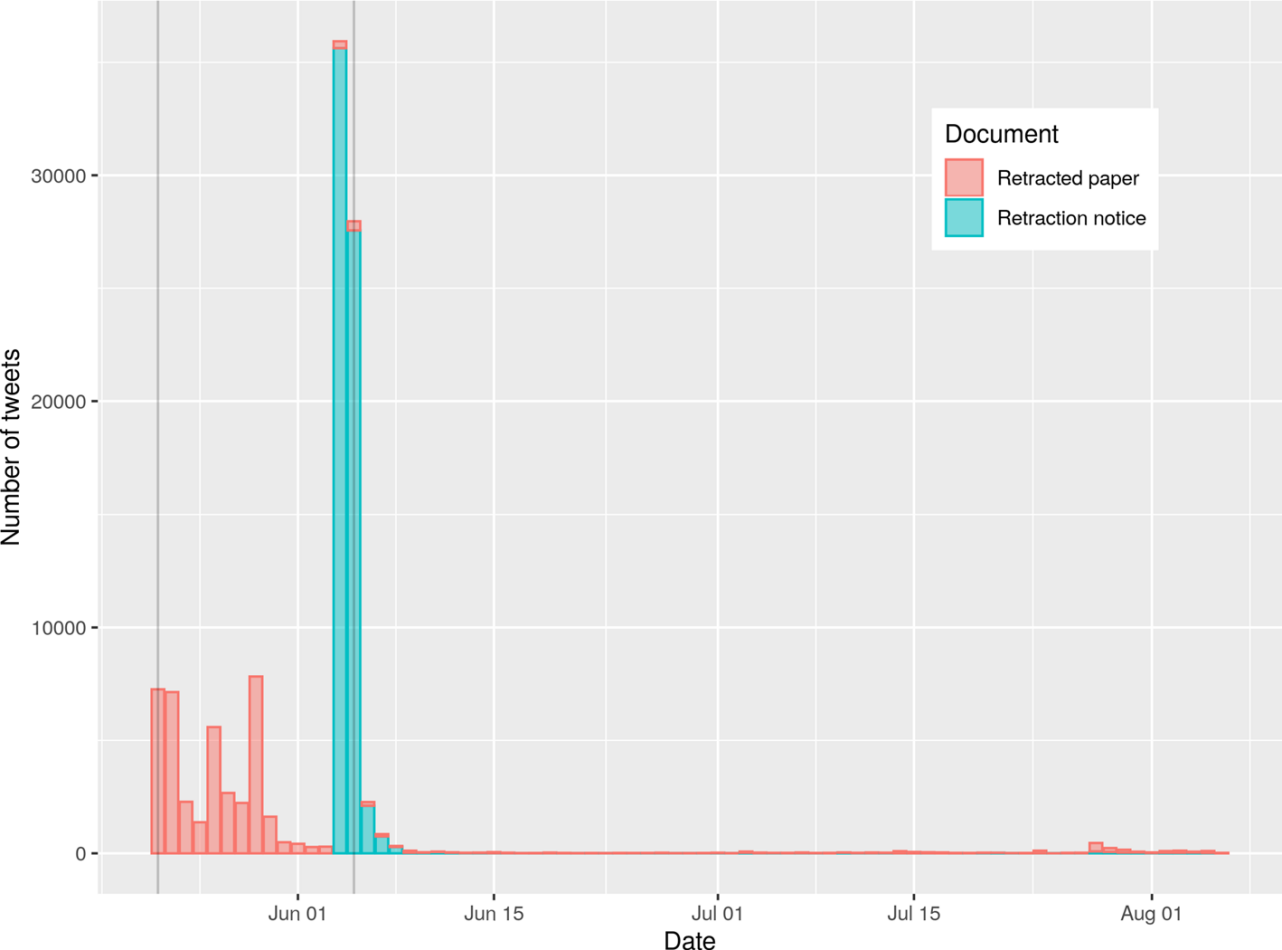
Tweets per day that mention either the paper Mehra et al., (2020a) or its retraction Mehra et al., (2020b) . The publication dates of the paper and the retraction notice are marked as gray vertical lines

Figure 9 shows the tweets per day that mention either the paper Mehra et al., (2020a) or its retraction Mehra et al. (2020b). The publication dates of the paper and the retraction notice are marked as gray vertical lines. The retraction notice has been tweeted a day before its official publication date. On these 2 days the most tweets per day were recorded for the retraction notice. Because the retraction notice has been tweeted a day before its official publication, the two time periods were organized slightly different: (i) before the day before the publication date of the retraction notice and (ii) since the day before the publication date of the retraction.

**Fig. 10:**
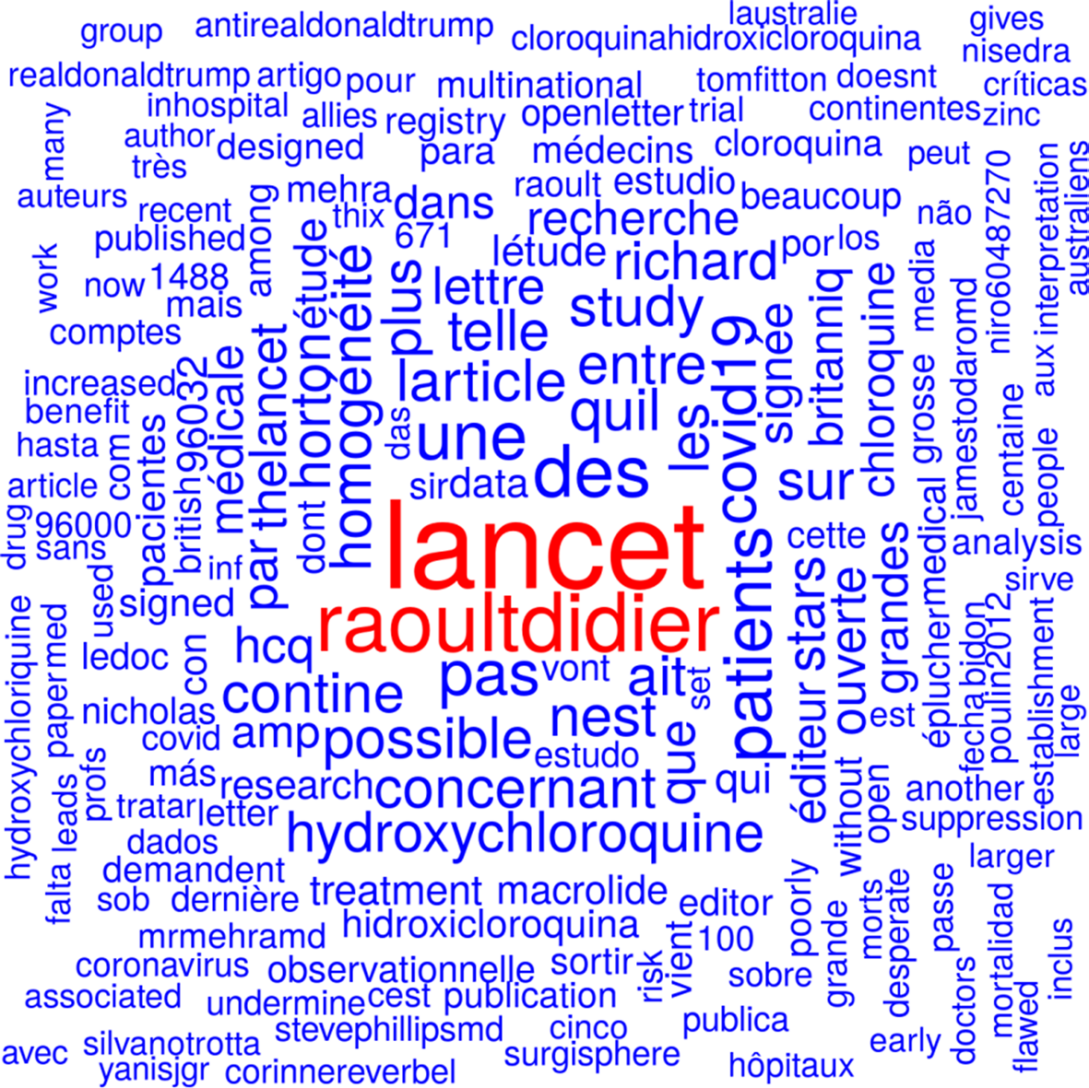
Word cloud from tweet texts based on the tweets mentioning Mehra et al., (2020a), before the day before the publication date of the retraction

**Fig. 11:**
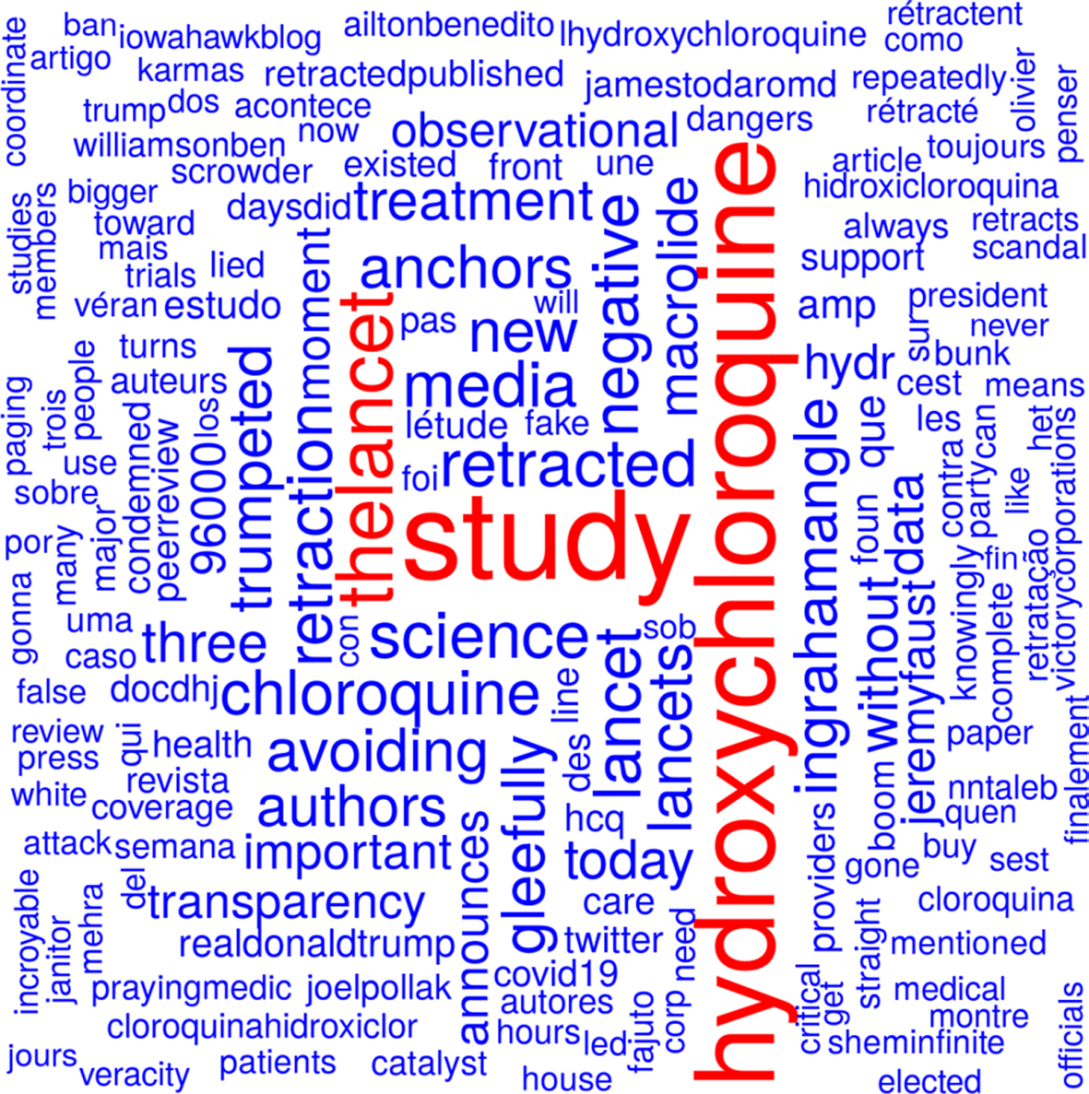
Word cloud from tweet texts based on the tweets mentioning Mehra et al., (2020a), or Mehra et al., (2020b) since the day before the publication date of the retraction

Figure 10 shows a word cloud from tweet texts based on the tweets mentioning Mehra et al., (2020a) before the day before the publication date of the retraction. Figure 11 shows a word cloud from tweet texts based on the tweets mentioning Mehra et al. (2020a), or Mehra et al., (2020b) since the day before the publication date of the retraction. The most prominent terms in Fig. 10 are ‘lancet’ (the journal the study was published in) and ‘roaultdidier’ (a French physician and microbiologist specializing in infectious diseases who promoted the hydroxychloroquine-based treatment of COVID-19). The most pronounced terms in Fig. 11 are ‘study’, ‘thelancet’, and ‘hydroxychloroquine’. The terms ‘retraction’ and ‘retracted’ are also quite prominent.

The publication Mehra et al., (2020a) was retracted because there was some doubt about the data basis from the company Surgisphere. The term ‘surgisphere’ appears at the lower border of Fig. 10. The term ‘data’ can be found in the right part of Fig. 11. The study Mehra et al. (2020a), was mentioned 39,477 times on Twitter before the retraction notice was tweeted for the first time (4 June, 2020). Out of these 39,477 tweets, 720 contained the term ‘surgisphere’ and 1819 contained the term ‘data’. The first ten tweets mentioning the study and using the term ‘data’ occurred on the day of publication of the study. None of them mentioned problems regarding the employed data. The first tweet mentioning the term ‘surgishphere’ was posted on the day of publication of the study (Stamets, 2020) but did not cast doubt on the validity of the data. A later tweet from 24 May 2020 (i.e., 2 days after publication and 11 days before retraction of the study) mentioning ‘surgisphere’ as a twitter handle (Arkancideisreal, 2020a) referenced a tweet that casted doubt on the validity of the data (Arkancideisreal, 2020b). Many later tweets mentioning the study and using the term ‘surgisphere’ posted between 29 May 2020 and 2 June 2020 also casted doubt on the employed data, see for example Pinjos (2020) and Schwartz (2020). Some of them linked to an open letter to the authors of the study and Richard Horton (Editor of *The Lancet*), see Watson (2020). Both terms, ‘data’ and ‘surgisphere’, occurred in 61 tweets.

## Discussion

Bornmann and Haunschild (2018) have shown in a case study that simple counting of mentions on Twitter for measuring impact or attention of science is problematic. Content assessment is important when Twitter data are used since wrong conclusions might be reached when simple mention counts are employed. Although cases of papers being retracted after publications are only ‘outliers’ in science, they are not the only cases that distort the validity of altmetrics data. Sugimoto (2016) mentions some others: “One can easily find examples of extremely high Altmetric.com scores which are the result of a viral joke, proofreading error, or scientific hoax. Behind these outliers are undoubtedly scores of articles whose recognition in policy documents, popular press, and on social media is a legitimate sign that the work is relevant and interesting to a broader public. How to identify the underlying mechanism of altmetric attention remains a critical challenge”.

Methodological mistakes, data errors, and scientific misconduct are prevalent problems in science that are often difficult to detect. In this case study, we are interested in the question whether tweets can be used to detect problems early with scientific papers. Since tweets are usually published very early after the appearance of a paper and some authors of tweets may have read the paper in detail, these authors may indicate the problems. We have analyzed three different papers about COVID-19/SARS-CoV-2 that were retracted at least 2 weeks after publication. We have analyzed the tweets per day since publication until August 2020 for the publications and their retraction notices. We have investigated the tweet texts with regard to the question whether the reason for retraction can be found in the tweets before or after retraction.

Mixed conclusions can be drawn from this case study. Our results show that not all problems of publications can be spotted using Twitter data. However, one can find hints to problems regarding publications in tweet texts. In the tweets that mentioned Bae et al., (2020a), we were not able to find early warnings regarding problems with the publication. Only a tweet on the night before the retraction occurred, was connected to the retraction reason. We were able to find early warning signs in tweet texts that mentioned the other two retracted publications of our study. However, the early warning signs from @John_Will_I_Am regarding the study by Wang et al., (2020a) are less compelling than the clear warnings from @Arkanicide_is_real regarding the study by Mehra et al., (2020a).

It is noteworthy, that tweet texts have to be analyzed because many tweets mentioning a publication are not a clear indicator of problems with a publication. Performing more and larger case studies like ours might yield a list of Twitter users who often mention problems with publications early on. Many case studies might lead in a meta-analysis to a curated list of Twitter users who often spotted problems in publications in the past. Such a curated list of Twitter users might be helpful for revealing problems in future publications earlier. Maybe also more problematic publications can be found with such a Twitter-based discovery system than without such a system. Besides monitoring the activity of special Twitter users, a search term list of alarming keywords might be helpful. Such a list will be subject-related and differ from one field to another. Experts of the fields should propose or at least check such a list. Limiting such monitoring to publications that are also discussed on post-publication peer-review sites, such as PubPeer, or in blogs might provide a more focused monitoring.

The current study only focuses on tweets of three publications and their retractions. Subsequent studies could extent the database by including a broad set of publications and by including non-retracted publications as a control group. It would be interesting to see whether our (mixed) results could be confirmed or not. If these studies demonstrate that tweet texts are a comprehensive source for indicating problems with publications, future studies could combine multiple warning signals for setting up a corresponding recognition system. Such automated systems can only provide hints. A careful check by experts in the field will be needed for verification or falsification of the suspicion of problems with publications.
